# Advancing justice and sustainability through health system resilience under economic sanctions

**DOI:** 10.1093/heapol/czaf091

**Published:** 2026-06-29

**Authors:** Reza Majdzadeh, Haniye Sadat Sajadi

**Affiliations:** School of Health and Social Care, University of Essex, Wivenhoe Park, Colchester, CO4 3SQ, United Kingdom; Knowledge Utilization Research Center, Tehran University of Medical Sciences, # 1547, North Kargar, 7th floor, Apt 7, 14179, Tehran, Iran; University Research and Development Center, Tehran University of Medical Sciences, # 12, East Nosrat, North Kargar, 14179, Tehran, Iran

**Keywords:** politics of health reform, theory of change, institutionalization, universal health coverage, health policy, Iran

## Abstract

Prolonged economic sanctions—often framed as non-military tools—have increasingly harmed health systems, especially where institutional and policy capacities are fragmented. Despite the growing debate on sanctions, documented analyses of health system responses remain scarce. This study examines Iran to assess how prolonged sanctions shaped its health policy architecture and resilience capacity, with attention to compounded crises such as COVID-19.

Phase one synthesized seven empirical studies conducted by the authors—including document reviews, interviews, Delphi, and policy analyses—to assess how resilience principles were embedded across the four stages of the health policy cycle: agenda-setting, policy formulation, implementation, and evaluation. These findings formed the foundation for Phase two, which applied an expanded Theory of Change (ToC) framework to reconstruct policy logics, surface implicit assumptions, and identify institutional breakpoints. After modelling the ToC, a panel of experts reviewed and validated the findings, ensuring methodological rigour and contextual accuracy in mapping resilience under sanctions.

The findings indicate that while Iranian health authorities implemented adaptive measures, responses were shaped by fragmented coordination, untested assumptions, and limited structured learning systems. Resilience limitations emerged during implementation and were embedded in early design phases, not fully anticipated or addressed, given the complex and uncertain policy environment.

This study offers an analytical framework for mapping resilience in policy systems under long-term constraints. Building on identified governance and design weaknesses, we recommend strengthening international legal safeguards; establishing protected humanitarian corridors; institutionalizing risk-informed planning; routine scenario-based resilience testing; and feedback-driven learning mechanisms within national policy systems. These capacities are essential to absorb shocks and enable adaptive, inclusive, sustainable responses. By clarifying structural domains where resilience can be embedded in advance, the analysis offers guidance for countries under comparable pressures to target strategies in governance, planning, and resource protection. This provides a transferable blueprint for strengthening justice-oriented health systems under long-term constraints.

Key messages

- Iran’s health system mounted significant adaptive responses to sanctions; however, enduring shocks of this magnitude demand a coherent, system-wide strategy to embed resilience throughout health policymaking.
- Designing policy under prolonged crises requires anticipatory thinking—identifying where core assumptions may collapse and incorporating real-time feedback mechanisms to maintain system responsiveness.
- Lasting resilience relies on institutionalizing risk-aware planning, structured learning systems, and adaptive governance as core features of health policymaking.
- Routine resilience testing and scenario-based planning can enhance preparedness, strengthen institutional capabilities, and sustain reform in politically and economically constrained settings.

## Introduction

In recent decades, health systems worldwide have faced a convergence of complex, interacting threats, including pandemics, political and economic instability, climate change, forced migration, and armed conflict, which have resulted in significant economic losses and a decline in human capital (Kanem et al. 2023). These interlinked disruptions compromise routine health system operations and constrain their capacity to respond effectively, equitably, and promptly to evolving health needs (Thomas et al. 2020). Consequently, the concept of health system resilience—the capacity to absorb, adapt, and recover from acute shocks and chronic stressors—has become an essential determinant of sustainability and performance at both national and global levels (Barasa et al. 2018, Forsgren et al. 2022).

Among the external stressors threatening health systems, economic sanctions remain under-examined in integrated policy frameworks despite their expanding scope in global politics. In particular, systematic and peer-reviewed analyses of how health systems respond and adapt to prolonged sanctions are notably scarce, leaving a significant gap in the evidence base for policy design. Unlike most systemic threats, sanctions are externally imposed, unilaterally or multilaterally, as instruments of political leverage (Beal 2021). While often described as non-military instruments, sanctions can exert wide-ranging and long-lasting effects on health systems by disrupting supply chains, weakening institutional performance, and exacerbating service inequities.

Although impacts vary by scope, duration, and enforcement, convergent evidence shows sanctions disrupt medical supply chains, compress fiscal space for health, degrade workforce capacity, and widen inequities—effects that can extend to higher all-cause mortality (Pinna Pintor et al. 2023, Sajadi et al. 2023 , 2024b, Yazdi-Feyzabadi et al. 2024). Evidence links sanctions to increased mortality among older adults and children (Rodríguez et al. 2025) and reduced life expectancy (Gutmann et al. 2021). From a proportionality perspective, legal and empirical analyses find that the humanitarian rights costs borne by civilians outweigh the political gains, casting doubt on the measures’ justification under international law (Steinbach et al. 2023). These concerns align with the United Nations Human Rights Council’s position that economic sanctions—referred to in Council resolutions as ‘unilateral coercive measures’—which undermine the enjoyment of human rights, are contrary to States’ obligations under international human rights law (United Nations Human Rights Council 2014).

A nuanced understanding of these impacts is essential to developing resilience-oriented strategies that safeguard equity and system sustainability amid prolonged political and economic stress. These inequities raise normative and ethical questions about justice, accountability, and responsibility in health policymaking under sanctions.

**Figure 1.**
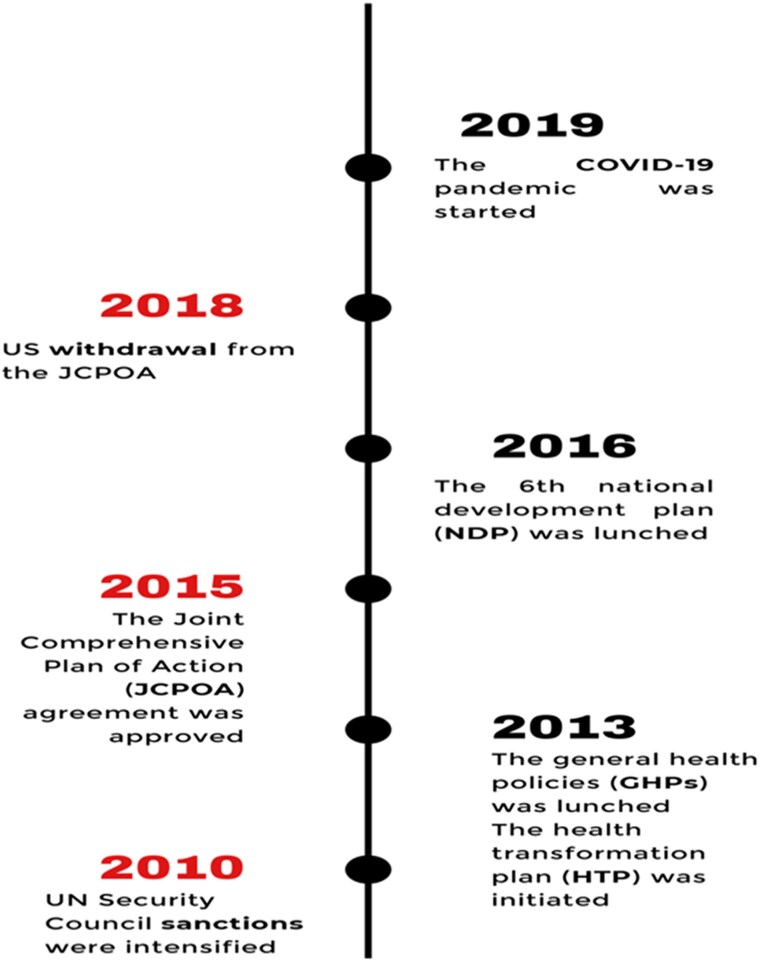
Timeline of major health policy milestones in Iran in relation to key sanctions events and the onset of the COVID-19 pandemic.

The Islamic Republic of Iran’s protracted exposure to sanctions, alongside other systemic shocks, offers a critical case for examining how external constraints reshape health policy design, resilience capacity, and equity outcomes. It has experienced multiple structural, political, and economic changes over the past four decades, including the 1979 revolution, the Iran-Iraq war (1980–88), and a long-standing regime of economic sanctions. Although initial sanctions date back to the early 1970s, they became formalized and escalated after the 1979 revolution and the 1979 events at United States of America (USA) Embassy (Aghazadeh 2013). Since the early 2000s, sanctions have intensified, particularly in response to Iran's nuclear programme. Notably, the escalation accelerated after 2010, as illustrated in Fig. 1, with a shift from multilateral UN Security Council resolutions to expansive unilateral measures by the USA, the European Union, and other actors.

The 2015 Joint Comprehensive Plan of Action (JCPOA) led to the temporary easing or suspension of several sanctions. However, the USA withdrawal from the agreement in 2018 resulted in a renewed and expanded wave of sanctions, affecting key sectors, including oil, banking, transportation, pharmaceuticals, and medical supplies (Danaei et al. 2019, Sajadi et al. 2021, Sajadi et al. 2023).

Over time, these measures have transitioned from tactical pressure tools into entrenched sources of socio-economic disruption, with particularly stark implications for Iran’s health system (Aghazadeh 2013). While the full extent of these consequences may not have been anticipated, evolving empirical evidence now calls for a fundamental recalibration of health policy frameworks to withstand protracted external constraints.

Fostering resilience under sanctions requires forward-looking, system-wide planning that moves beyond reactive or fragmented interventions. In this context, applying the Theory of Change (ToC) offers a valuable framework for reconceptualizing resilience—not as short-term responsiveness but as an ongoing process of institutional adaptation and learning (Rogers 2014, Mayne 2015, Paina et al. 2017). Defined broadly, a ToC is a structured planning and evaluation methodology that articulates long-term goals and maps the intermediate steps, assumptions, and preconditions required to achieve them. Given the complexity and non-linearity of health system shocks—such as overlapping sanctions and pandemics—the ToC framework is particularly suited for identifying assumptions, causal pathways, and systemic gaps (World Health Organization 2024). For countries like Iran, where exposure to sanctions has been both prolonged and intensifying, health policy could benefit from being deliberately structured to foster anticipatory and adaptive capacities that can respond to long-term geopolitical constraints.

While many studies evaluate health policymaking initiatives, few examine how resilience has been systematically embedded in health policymaking. This study addresses that gap by analysing key health policies and programmes implemented in Iran between 2014 and 2025 (1393–1403 SH) (Fig. 1). The General Health Policies (GHPs), ratified as part of Iran’s long-term strategic vision, articulate the country’s overarching health values, priorities, and policy directions presented in Fig. 1. Complementing this high-level framework, the Health Transformation Plan (HTP), launched in 2014, served as Iran’s flagship reform initiative to improve access, equity, and financial protection through a suite of structural interventions. In parallel, the Sixth Five-Year Economic, Social, and Cultural Development Plan [the Sixth National Development Plan (6th NDP)] set out the health sector’s obligations over a five-year horizon, providing a legal and planning framework to translate strategic aims into actionable commitments.

By addressing this gap, the study not only reconstructs Iran’s policy pathways under sanctions but also identifies transferable strategies for governance, planning, and resource protection that could inform countries facing similar constraints. It investigates how prolonged sanctions have shaped health policy responses aimed at resilience and assesses the extent and nature of institutional adaptation. It further evaluates whether these responses have mitigated or exacerbated health inequities, offering transferable insights for advancing justice and sustainability in similarly constrained settings.

## Materials and Methods

This study employed a structured, two-phase policy research design to examine how Iran's health system responded to prolonged sanctions and whether the strategies adopted contributed to institutionalizing resilience across policy domains. The study was grounded in a ToC framework and combined document analysis, qualitative policy evaluation, and interpretive reconstruction of causal policy pathways. This approach is particularly suited for complex, protracted crises such as sanctions, where causal assumptions, pathways, and systemic vulnerabilities require iterative examination.

**Figure 2.**
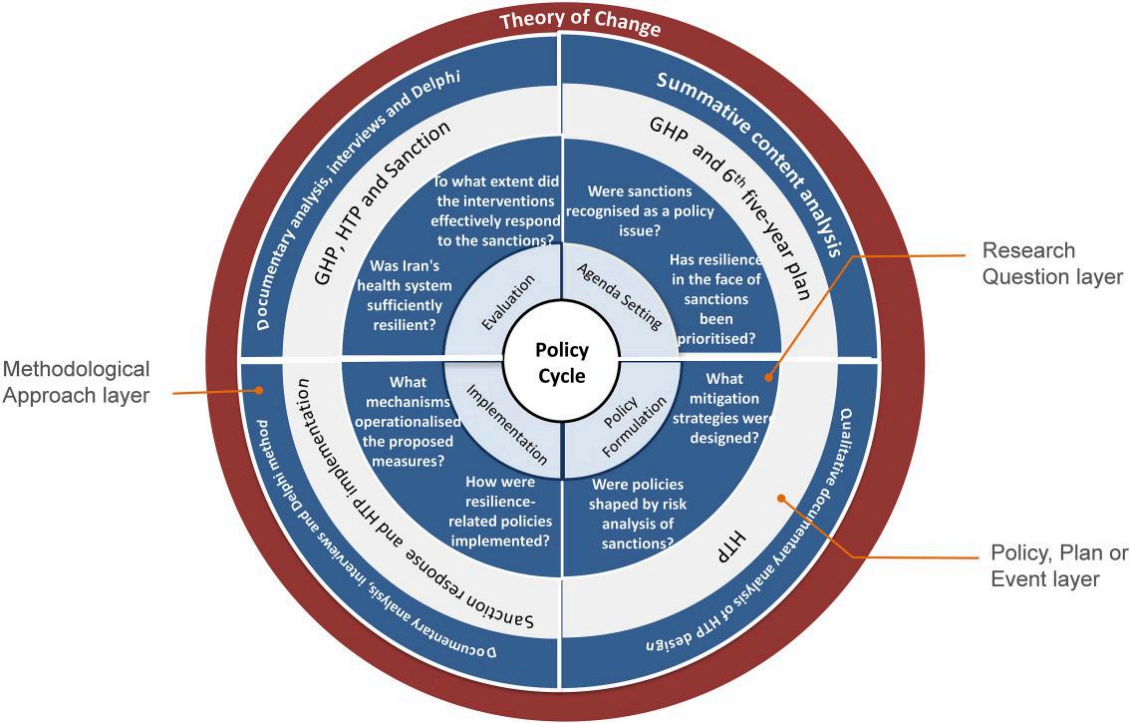
Two-phase study design: alignment of the health policy cycle with research questions, key policies and events, and underlying methodologies from Phase one.

**Figure 3.**
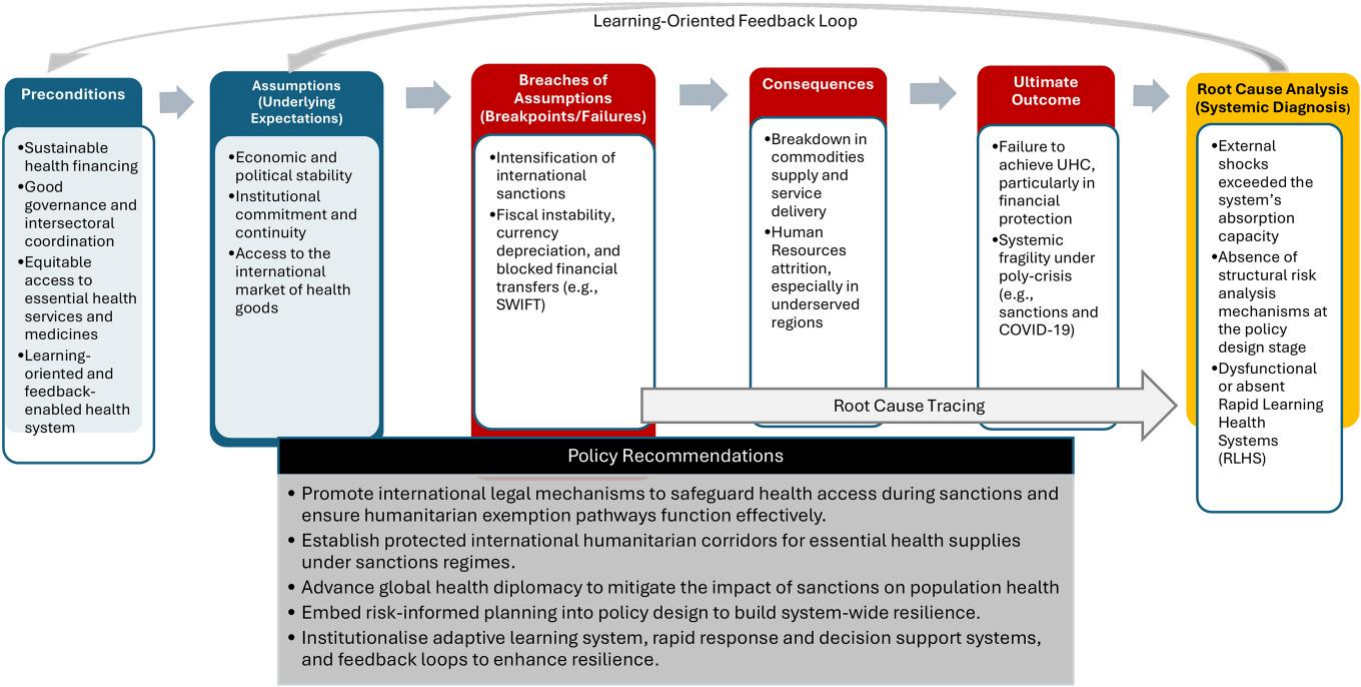
Phase two results: mapping of resilience gaps and policy breakdowns using a theory of change (ToC)†.† Visual layout is linear for clarity. The analytical process was non-linear and iterative, with breakpoints and root causes identified through ToC mapping and validated by expert review. A learning-oriented feedback loop illustrates how systemic insights from root cause analysis can inform the reassessment of initial assumptions and preconditions.

Figure 2 maps the study’s architecture, linking a stage-based health policy analysis to a ToC framework. The inner circle shows the four policy cycle stages—agenda-setting, policy formulation, implementation, evaluation—each paired with key analytical questions on sanctions and resilience. The second ring connects these questions to the documents and events analysed, while the outer ring identifies the methodological tools: document review, interviews, and Delphi rounds. The surrounding ToC layer frames the entire inquiry and is operationalized in Phase two (see Fig. 3).

### Phase one: policy analysis based on the policy cycle

This phase applied the policy cycle model—agenda-setting, policy formulation, implementation, and evaluation—to examine how sanctions were acknowledged and addressed in key national health documents. For each stage, essential questions were posed regarding how sanctions were identified, defined, and responded to, and relevant documents, events, and appropriate methods were selected to address them. The empirical foundation of this phase draws on seven primary empirical studies previously conducted and co-authored by the authors: content analysis of the GHPs (Sajadi et al. 2020), review of the 6th NDP (Mahdavi and Sajadi 2021), the Iran HTP design (Harirchi et al. 2020) and evaluation (Sajadi et al. 2019, World Health Organization 2019), assessment of sanction-related responses (Sajadi and Majdzadeh 2022), and analysis of GHPs implementation (Sajadi et al. 2024a). Most of these studies were published as independent peer-reviewed articles (Table 1). These prior findings were re-analysed and systematically mapped onto the Phase one research questions (Fig. 2), forming the empirical base for the ToC-informed synthesis in Phase two.

**Table 1.** Overview of primary studies supporting the empirical basis (the studies are ordered according to the stage of the policy cycle in which they are used).

Title	Year	Aim	Methodology	Main findings
Identifying National Health Priorities: Content Analysis of the Islamic Republic of Iran's General Health Policies (GHPs)	2020	To identify concepts and interventions formulated in the GHPs and to advance understanding as to what extent GHPs are aligned with global priorities.	Qualitative documentary analysis of 14 GHP articles using summative content analysis to quantify key concepts and identify themes. Iterative coding and categorization led to theme development; initial coding by one author, refined by team consensus. Expert panel (*n* = 23) reviewed and ranked categories/themes; consensus threshold: ≥ 75% approval Final themes were compared with universal health coverage (UHC) frameworks for alignment with international directions.	65 codes were categorized into four key themes, including social values; corrective measures; strategic orientations; vision Values encompass two categories: equity and professionalism. Equity was emphasized more by the GHPs. Corrective measures were divided into six categories, including strengthening the governance arrangements; improving delivery arrangements and implementation; reforming financing arrangements; introducing innovations in medical education and research; making system resilient; empowering and engaging with the community The ultimate goals comprised three categories, including health outcomes, health system performance, and country development The Iranian health system vision was to be one of the best-integrated health-care systems with medical education in the Western South Asia region and the Islamic world. In line with global health strategies, GHPs offer a country-specific map to accelerate progress toward sustainable development.
Qualitative analysis of the Iranian Sixth Five-Year Economic, Social, and Cultural Development Plan of the Islamic Republic of Iran (6th NPD) from UHC	2021	To analyse the 6th NPD to shed light on how the plan addresses the UHC	Qualitative content analysis of ‘Secs. 14—Health, Insurance, Health and Women, and Family in the 6th NPD Text was coded into meaning units, guided by the WHO Six Building Blocks of Health System and UHC principles.	Six themes and 21 subthemes identified via content analysis, including financing (fair resource pooling, payment methods, revenue generation, basic benefits package); governance and leadership (integration of social insurance, provider compliance, Ministry of health as regulator, payer-provider split, stakeholder participation); health workforce (align workforce quality and quantity with health needs); health information systems (electronic health records, delivery and financing information systems); organization and delivery (improve delivery efficiency, strengthen family physician and referral system, enhance pre-hospital emergency care); access to medicine (develop essential drug list, ensure generic medicine coverage and provision)
How health transformation plan (HTP) was designed and implemented in the Islamic Republic of Iran?	2020	To examine how historical deficits and political changes in Iran enabled the launch of the Health Transformation Plan (HTP), and to assess its interventions, short-term achievements, and impacts on health system functions and performance goals.	Qualitative documentary analysis of validated key HTP-related documents. Kingdon’s Multiple Streams framework was used to analyse HTP’s policy agenda emergence Control Knobs framework applied to evaluate HTP-induced system changes Latent content analysis was employed for data interpretation.	HTP addressed both historical deficits and urgent challenges in Iran's health system including weak Ministry of Health regulation and largely unregulated private sector; high out-of-pocket payments (>50%) and regressive insurance contributions; fragmented insurance funds and delayed reimbursements leading to informal payments; inefficient provider payment methods; lack of care coordination and clinical guidelines; unequal resource distribution and poor use of health technology assessments; economic sanctions from 2011 onward causing reduced government revenue, deprioritized health funding, inflation, and currency crisis. Policy window opened with the Supreme Leader's GHPs and strong parliamentary support. A national committee was formed in 2013 to address the urgent medicine crisis. Key outcomes included reduced OOP expenditures through new financing mechanisms and safety nets; improved service provision via workforce recruitment, facility development, and PHC expansion to urban/peri-urban areas; positive impacts on access, quality, and patient satisfaction. HTP is recognized as a health system reform, though further agenda-setting is needed to enhance value for money in health spending.
Universal health coverage in Iran: where we stand and how we can move forward	2019	To introduce Iran's latest fundamental plan for achieving UHC, outline the main challenges, and propose practical interventions to address current problems.	Desk review	Key UHC challenges include resource sustainability, updated service delivery, and strong governance. Addressing them requires system-thinking-based interventions and a strong national commitment to institutionalize change.
Health system transformation in the Islamic Republic of Iran: An assessment of key health financing and governance issue	2019	The World Health Organization's publication provides a comprehensive evaluation of Iran’s health system reforms, particularly in the context of the 2014 HTP. This assessment focuses on critical aspects of health financing and governance, offering insights into the country’s efforts to progress toward UHC. Key highlights: Historical Context: Iran’s health system has been recognized for its robust primary health care (PHC) infrastructure, established in the 1980s, which laid the groundwork for subsequent reforms. HTP: Launched in 2014, the HTP aimed to address systemic challenges, including high out-of-pocket expenditures, fragmented insurance schemes, and inefficiencies in service delivery. Financing Reforms: The report examines efforts to enhance financial protection, such as expanding insurance coverage and reducing patient cost-sharing, to alleviate the economic burden on households. Governance Enhancements: It analyses initiatives to strengthen health sector governance, including institutionalizing public participation platforms and improving regulatory mechanisms. Policy Recommendations: Based on the assessment, the publication proposes policy options to advance Iran’s journey toward UHC further, emphasizing the need for sustainable financing and robust governance structures.		
Health system to response to economic sanctions: global evidence and lesson learned from Iran	2022	To identify and propose a set of mitigating measures and response strategies to improve Iran’s health system performance in the face of sanctions	Three-stage study in Iran (2020–2021): Rapid review of evidence on health system resilience measures under sanctions. Qualitative phase using semi-structured interviews and document analysis with 10 experts to explore mitigation measures. Two-round Delphi with 11 experts to reach consensus and prioritize mitigation measures.	62 mitigation measures extracted from 13 studies; 18 additional measures identified via expert interviews. Measures categorized into five themes, including sustained financing, good governance, updated health information systems, qualified workforce, and equitable service delivery. In Delphi Round 1, 28 measures identified; 9 prioritized as highly effective and feasible, including proactive inventory control; national essential medicines list, clarified use of oil revenues for medicine procurement; tailored service packages for vulnerable groups; efficient surveillance system; lower imported medicine prices; dual equity-priority policies; fair resource allocation; clinical guidelines provision
Progress toward the implementation of GHPs in Iran	2024	To assess progress in implementing Iran’s GHPs, demonstrate effective monitoring/evaluation of national plans, and identify barriers to full implementation.	Available data sources, formal reports, and studies were examined to gather data on selected indicators. Documentary analysis and 21 semi-structured interviews were conducted to identify measures taken to materialize IGHP Data were analysed using the content analysis method.	Several indicators showed improvement over the study period; data were missing for some indicators Barriers to Iran's GHP implementation included: incomplete understanding of policies; lack of necessary mechanisms and infrastructure; poor alignment and coherence of national health plans and policies; absence of a monitoring and evaluation framework; weak transparency and accountability in the health system Lack of clarity on progress remains a concern for ongoing health plan development

This analysis explored whether resilience was intentionally embedded into the policy architecture or emerged as a reactive response to crises—recognizing that policymaking in constrained settings often unfolds under dynamic and uncertain conditions. The data collected in Phase one were synthesized using the ToC framework to map and examine the causal pathways that shaped health policy outcomes under sanctions.

### Phase two: learning and adaptation using the theory of change

Findings from Phase one were systematically synthesized to inform the expanded ToC analysis in Phase two, ensuring a direct link between empirical evidence and causal pathway reconstruction. It corresponded to the section marked in red in Fig. 2, where findings from the policy cycle analysis (Phase one) were synthesized by the authors using an expanded ToC framework. The objective was to identify systemic factors that undermined the achievement of policy objectives, particularly under the prolonged pressures imposed by sanctions.

The analytical approach moved beyond conventional linear ToC models by integrating two additional components:

- Breakpoints—critical junctures where expected causal linkages between policy elements failed to materialize.
- Root-cause analysis, which explored why these breakdowns occurred and what systemic factors contributed to them.

The choice of this framework was informed by established evaluation scholarship. Mayne emphasizes the need for transparent assumptions and testing the strength of causal chains in complex interventions (Mayne 2015). Breuer et al. highlighted the importance of linking preconditions, feedback mechanisms, and performance indicators in evaluating public health systems (Breuer et al. 2016). Rogers underscores the value of identifying implicit assumptions and mapping potential failure points to inform adaptive policy management (Rogers 2014).

Operationalization of the analytical framework proceeded through four defined steps:

- Causal mapping: Policy documents were deductively coded against a logic model linking intended outcomes with necessary preconditions, assumptions, interventions, and indicators. Components that were missing or undefined were noted as latent nodes—often reflecting contextual unpredictability rather than intentional omission.
- Breakpoint identification: The reconstructed causal map was cross-checked against empirical data from Phase one to identify breakpoints. Where critical requirements were either missing from the policy texts or not realized in practice, these gaps were classified as latent nodes or breakpoints.
- Root-cause analysis: For each breakpoint, contributory factors were traced using iterative ‘why–why’ questioning, drawing on interview findings, routine data, and document analysis.
- Validation and plausibility testing: Five independent health policy experts reviewed the reconstructed causal pathways and associated root-cause narratives. Competing interpretations were either incorporated or documented as analytical limitations. This validation ensured that conceptual interpretations were both theoretically sound and practically relevant to policy contexts under sanctions.

The expanded ToC framework retained the structural clarity of conventional models while adding diagnostic depth to capture how systemic shocks—such as sanctions and the COVID-19 pandemic—disrupted intended policy outcomes. Although the framework followed a phased design, its analytical process was inherently non-linear. Key concepts such as breakpoints and root causes were not predefined but inductively derived from recursive interrogation of Phase one data, guided by theory and empirical irregularities. The analysis iterated between documentary evidence, interview insights, and theoretical constructs, with continuous validation by independent experts. This adaptive reasoning enabled the reconstruction of disrupted policy logics that would be overlooked in linear models of evaluation.

## Results

This section presents the study’s findings across two phases.

### Phase one: policy analysis based on the policy cycle

This phase traced how Iran’s health system recognized and responded to sanctions across the four stages of the policy cycle—agenda-setting, policy formulation, implementation, and evaluation. Guided by stage-specific analytical questions (see inner layer of Fig. 2), we revisited primary studies and policy documents to generate the empirical base for Phase two’s causal reconstruction.

Agenda-setting: This stage assessed whether sanctions were explicitly identified as systemic risks in strategic health documents and whether institutional resilience was positioned as a policy priority. The absence of such framing is notable, considering that both the GHPs and 6th NDP were developed amid intensified economic sanctions—suggesting a missed opportunity to institutionalize risk awareness at the agenda-setting level.

In the content analysis, the GHPs were categorized into four thematic pillars: social values (e.g. equity and support for vulnerable groups), reform strategies (including governance improvement and financial reforms), strategic orientations (e.g. strengthening system performance), and long-term aspirations (e.g. positioning Iran as a regional scientific leader in health). The 6th NDP further outlined six core domains: financing (resource pooling, payment reforms, and a basic benefit package), governance (e.g. insurance integration and regulatory authority of the Ministry of Health), human resources (alignment with system needs), health information systems (e-health records and monitoring tools), service delivery (e.g. family physician and pre-hospital emergency care), and pharmaceuticals (e.g. essential medicines list and rational prescribing).

Where present, resilience-related measures largely reflected generic system-strengthening language and lacked tailored strategies for sanction preparedness. This suggests sanctions were institutionally underestimated as structural risks, entrenching subsequent policy vulnerability.

Policy formulation: This phase was guided by two questions: whether sanctions were explicitly considered in the design of health sector reforms, and whether resilience-oriented measures were embedded in their formulation. To explore these questions, we analysed the HTP, launched in 2014 shortly after the GHPs, as the flagship reform initiative of the period (Harirchi et al. 2020).

Evidence from prior studies indicates that the HTP emerged amid converging financial pressures, rising treatment costs, and increasing public expectations—all exacerbated by international sanctions. The launch coincided with declining oil revenues, worsening currency instability, and surging prices of medicines and medical supplies. The HTP aimed to achieve universal health coverage (UHC) and reinforce the health system through five goals: sustainable financing, financial protection, improved access, quality enhancement, and multisectoral engagement. Interventions included expanded insurance funding, price controls, reduced out-of-pocket expenses, and support for domestic pharmaceutical production—most implicitly relevant to resilience but not framed as such. However, the design was more reactive to immediate political pressures and public demands than grounded in a resilience-oriented logic, relying on untested assumptions about funding continuity, insurer coordination, and political stability, without formal risk appraisal. Despite a ‘window of opportunity’ to integrate sanctions into health policymaking, the HTP contained no specific interventions or contingency planning for such shocks.

Policy implementation: This examined how the rollout of the HTP coincided with escalating international sanctions, and how Iran’s health system attempted to pursue internal reform while responding to external shocks. To address the central question of what concrete measures were taken to mitigate sanctions, we analysed two evaluation reports: one on the HTP (Sajadi et al. 2019, World Health Organization 2019) and another on broader health system responses to sanctions (Sajadi and Majdzadeh 2022).

The World Health Organization (WHO) reported initial gains; however, following reimposition of sanctions—particularly after the U.S. withdrawal from the JCPOA in 2018—access to essential foreign currency and financial channels became severely restricted. These restrictions directly affected imports of medical equipment, pharmaceuticals, and hospital supplies, disrupting several HTP service packages. The government adopted a series of reactive, short-term measures, largely reliant on domestic capacity and improvisation.

Sanction-related actions spanned three primary domains:

1. Pharmaceuticals and medical equipment: Sanctions severely disrupted supply chains for high-cost and import-dependent medicines, particularly for cancer, transplant, and rare disease treatments. In response, the government formed a National Drug Supply Committee, prioritized foreign currency allocation, revised the national drug list, and promoted domestic pharmaceutical production. Nonetheless, critical shortages persisted—particularly among vulnerable patient groups—underscoring the limitations of domestic substitution strategies.
2. Financial and managerial interventions: Emergency foreign exchange reserves were mobilized, tariffs on essential equipment were reduced, customs exemptions were applied, and scarce resources were redistributed. In parallel, selected health authorities—medical universities—developed internal protocols to promote rational drug prescribing and mitigate local shortages.
3. Institutional mechanisms: Temporary crisis units, such as the Special Pharmaceutical Taskforce, improved intersectoral coordination during acute sanction pressures but lacked permanence—operating without mandates, timelines, or integration into routine structures—underscoring the reactive nature of the policy environment.

During implementation, the health system’s response to sanctions was largely improvised and short-term, lacking structural foresight or integration into existing policy frameworks. This absence of institutionalization, predictability, and systemic adaptability undermined sustainable resilience. Persistent health-workforce attrition—especially in underserved areas—and chronic supply chain disruptions exposed the fragility of core implementation assumptions. In the end, the inability to secure essential medicines and maintain frontline capacity revealed deeper governance and planning deficiencies, with disproportionate impacts on vulnerable populations.

Policy Evaluation: This stage assessed the extent to which Iran’s health system demonstrated resilience under sanctions and evaluated the effectiveness of implemented interventions. Two key studies—evaluating the GHPs (Sajadi et al. 2024a) and the HTP (Sajadi et al. 2019, World Health Organization 2019)—served as benchmarks for outcome assessment. The GHPs review found that, despite advances in infrastructure expansion and insurance coverage, critical areas such as governance, intersectoral collaboration, and performance oversight were largely neglected. Resilience-related reforms were either poorly executed or lacked measurable evaluation criteria. Key indicators—including health spending as a percentage of GDP (Gross Domestic Product)and the effectiveness of fiscal interventions—showed declining trends or were undermined by data limitations.

Implementation barriers included limited understanding of policy content, inadequate operational capacity, weak alignment among national health programmes, absence of structured evaluation frameworks, and restricted transparency and accountability. These constraints hindered the sustainability of financing, service delivery, and governance. Recommendations emerging from the evaluations included revising payment models and strengthening institutional oversight. Collectively, the findings point to a critical weakness in Iran’s policy feedback loop—reflecting not only a lack of evaluative foresight but also a missed opportunity to institutionalize organizational learning for future resilience planning.

In sum, Phase one revealed three systemic deficits: (i) the absence of embedded mechanisms for continuous policy evaluation and revision; (ii) weak structural foundations, including insufficient intersectoral coordination and a lack of institutionalized policy knowledge systems; and (iii) gaps in implementing resilience-oriented interventions that remaining fragmented and unanchored in broader system strategies. These weaknesses were further exposed under the compounded pressures of sanctions and the COVID-19 pandemic, which intensified systemic vulnerabilities—particularly in achieving UHC and maintaining equity in service delivery (Takian et al. 2020).

Collectively, the evidence illustrates a fragmented and reactive approach to resilience—marked by disconnected interventions that lacked integration into a unified strategy for absorbing or adapting to sustained external shocks. Table 2 synthesizes these findings by mapping responses to eight policy cycle questions across the four policy stages. Building on these findings, Phase two of the study moved beyond descriptive stage-based analysis to examine the underlying causal logic of policy successes and failures. By applying an expanded ToC framework, we traced how intended policy outcomes diverged from actual system responses—revealing hidden assumptions, critical design flaws, and structural breakpoints.

**Table 2.** Summary of analytical questions and Phase one findings based on the health policy cycle.

Policy stage	Analytical question	Main finding
Agenda-setting	Were sanctions recognized as a policy issue?	Neither of the upstream policy documents made explicit reference to sanctions.
	Was resilience to sanctions placed on the policy agenda?	There were indirect or general references to strengthening resilience, but no specific design or action.
Policy formulation	Were sanctions considered in the design of health sector reforms?	The HTP design showed no specific interventions targeting sanctions; key assumptions lacked risk analysis.
	Were resilience principles embedded in policy design to address sanctions?	Most measures appeared reactive, not grounded in structural or resilience logic.
Policy implementation	What concrete measures were taken to address sanctions?	Multiple interventions were undertaken, but they were mostly short-term and reactive.
	Did these measures lead to structural resilience?	The absence of institutionalization, predictability, and systemic adaptability prevented sustainable resilience.
Policy evaluation	Did Iran’s health system achieve any level of resilience to sanctions?	The available evidence suggests limited progress in building structural resilience. During poly-crises (sanctions and COVID-19), multiple system constraints were reported.
	Were the interventions undertaken to address sanctions effective?	There was no systematic evaluation of effectiveness. Feedback and organizational learning mechanisms were largely absent.

### Phase two: learning and adaptation using the theory of change

Phase two applied a causal lens using an expanded ToC to integrate insights across all stages of the policy cycle and identify the structural mechanisms and institutional dynamics that enabled or constrained resilience (Fig. 2). The objective was to explain why key strategic reforms—particularly the GHPs and the 6th NDP—either failed to recognize sanctions as systemic shocks or to embed adaptive mechanisms within policy architecture.

As depicted in Fig. 3, the colour scheme clarified the logic path for readers: blue denotes Preconditions and Assumptions (foundational expectations), red marks Breakpoints, Consequences, and Ultimate Outcomes (effects of disruption), and yellow highlights Root-Cause Analysis (diagnostic insights informing adaptation). This coding aligned analytical components with their roles in the causal chain and makes points of failure and learning visually explicit. This design choice visually reinforced the recursive analytical structure and the feedback flow into earlier stages of the causal chain.

We developed a structured matrix to align the key ToC components—goals, preconditions, interventions, assumptions, and indicators—with Phase one findings. Figure 3 organized results into seven interdependent components, reflecting the reconstructed causal pathway. While the diagram adopts a simplified left-to-right layout, the analytical logic was non-linear and recursive.

Key categories—such as breakpoints and root causes—were derived from iterative synthesis of Phase one data and cross-checked against expert feedback.

1. Preconditions: Realizing long-term health goals necessitated foundational conditions such as sustainable financing, robust governance, uninterrupted access to essential services, and functioning learning systems [e.g. Rapid Learning Health Systems (RLHS)—which integrate real-time evidence into decisions (Ahmadi et al. 2024)]. These enablers were often either absent from key policies or inadequately operationalized.
2. Assumptions: Policy formulation rested on assumptions—such as macroeconomic stability, institutional continuity, and international market access—that ultimately proved untenable under dual shocks of sanctions and COVID-19, revealing fragile foundations in strategic planning.
3. Breaches of assumptions: Key disruptions included the intensification of international sanctions and resulting fiscal instability, currency depreciation, and blocked financial transfers. These shocks revealed a growing misalignment between policy intent and systemic capacity, limiting the health system’s ability to adapt.
4. Consequences: The immediate consequences included breakdowns in commodity supply and service delivery, alongside health-workforce attrition—particularly in underserved regions. These outcomes intensified access inequities and contributed to a decline in public trust.
5. Ultimate outcomes: The cumulative result was a shortfall towards UHC—especially in terms of financial protection. The health system exhibited systemic fragility under the pressures of multiple concurrent crises, such as sanctions and the COVID-19 pandemic.
6. Root cause analysis: The analysis identified gaps in risk diagnostics during policy design and the absence or dysfunction of RLHS. Severe external shocks exceeded the system’s absorptive capacity.
7. Policy recommendation: The analysis identified several persistent structural weaknesses constraining resilience: lack of risk-informed planning, weak institutional learning systems, fragmented crisis coordination, and insufficient international protections for health in sanctioned settings. These limitations were most evident in disrupted access to medicines, fragmented governance, and short-term reactive planning.

The study identified five core areas requiring policy attention at both national and international levels, which are conceptually mapped in Fig. 3 and elaborated further in the next section.

## Discussion

This study applied a structured, two-phase analysis to assess how, and to what extent, resilience principles were integrated into Iran’s health policy processes. Combining the health policy cycle with a ToC framework, the analysis mapped institutional assumptions, identified causal gaps, and examined policy logic to clarify how long-standing institutional shortcomings—many predating recent crises—constrained system resilience. Drawing on findings from both phases, this section critically examines how high-level policy goals were, or were not, translated into effective institutional mechanisms and practice.

Iran’s overarching health policy documents, particularly the GHPs, articulate commitments to equity, accountability, and the pursuit of UHC (Sajadi et al. 2020). The HTP was similarly introduced to address financial gaps and reduce inequalities in access and service delivery (Sajadi et al. 2019, Harirchi et al. 2020). However, evidence indicates that sanctions undermined health infrastructure, fiscal stability, and the availability of qualified human resources—while exacerbating inequalities in access to care (Sajadi and Majdzadeh 2022). These pressures were further compounded by the COVID-19 pandemic, which significantly constrained the system’s capacity to implement even existing policies effectively (Karimi and Turkamani 2021). International organizations have repeatedly noted that economic sanctions on Iran hinder equitable access to essential health services (Smith 2020).

The analytical framework revealed how institutional voids and flawed causal linkages compromised the effectiveness of health policies amid this multifaceted crisis context. A central finding of this study is that broad structural weaknesses in policy design and governance undermined the ambitious goals outlined in Iran’s GHPs and the 6th NDP. While the scale of future shocks could not have been entirely foreseen in 2013, the lack of fiscal space analysis and the absence of contingency planning in major national strategies—despite the escalation of sanctions since 2010—indicates significant foresight failures. These gaps were not merely regrettable omissions; they constituted avoidable vulnerabilities in light of the available evidence at the time. The analytical framework traced these failures across multiple layers—ranging from ignored prerequisites (e.g. sustainable financing) to untested assumptions (e.g. political stability), and the absence of mechanisms for feedback and learning. This suggests that failure stemmed less from implementation weaknesses than from systemic flaws embedded in policy design, assumption-setting, and feedback architecture across the policy cycle. Had these reforms been implemented in a more stable economic environment, outcomes may have differed—but the design vulnerabilities would likely have persisted. These findings reinforce the importance of anticipatory policy design and institutional adaptability as strategic imperatives for health systems operating under structural constraints.

We acknowledge that when structural threats—such as conflict or sanctions—are politically sensitive, health policymakers may have limited autonomy to recognize or address these challenges beyond the boundaries set by national political discourse. Rather than assuming ideal conditions, this analysis seeks to identify pragmatic and feasible strategies within politically constrained and resource-limited settings. Disaggregating these shortcomings across the stages of the policy cycle allows for a more granular understanding of how and where institutional failures emerged.

At the ‘agenda-setting phase’, upstream policy documents—such as the GHPs and the 6th NDP—did not incorporate a structural analysis of economic sanctions. Our analysis confirms that sanctions were neither formally recognized as a policy issue nor integrated into the agenda-setting process—revealing a critical blind spot in national risk identification. This omission hindered the development of evidence-informed interventions to address emerging crises.

This understanding aligns with recent syntheses that conceptualize resilience not as simple persistence, but as a dynamic, capability-oriented property—adaptive and equity-attentive in nature. As Witter et al. argue, when policies overlook the origins and dynamics of systemic shocks during the agenda-setting phase, they are likely to replicate vulnerabilities throughout the entire policy cycle (Witter et al. 2023). The OECD Health Division similarly emphasizes the importance of agenda-setting in shock preparedness, noting that its absence often results in fragmented, short-term responses (Zimmermann et al. 2024). Al Asfoor et al. emphasize the importance of ‘resilience-enabling preconditions’, such as intersectoral coordination, accountable governance, and frontline workforce motivation—conditions largely absent from Iran’s early policy diagnostics (Al Asfoor et al. 2024). Building on this literature, we adopt an intersectionality-informed reading of resilience in sanctioned settings: not only the capacity to adapt, learn, and evolve, but also to do so in ways that recognize heterogeneous vulnerabilities and prevent ‘maladaptive’ adaptations that shift burdens onto already marginalized groups. Accordingly, resilience in this study is treated as a system capability that is both adaptive and equity-attentive, integrating preparedness (Zimmermann et al. 2024), institutional preconditions (Al Asfoor et al. 2024), and distribution-sensitive design (Vedadhir et al. 2025) rather than functional persistence alone. Taken together, our use of resilience both confirms and extends the prevailing literature—confirming the view of resilience as a dynamic capability (Witter et al. 2023, Al Asfoor et al. 2024, Zimmermann et al. 2024), while extending it by foregrounding intersectionality under sanctions (Vedadhir et al. 2025).

Applied to the Iranian context, this conceptualization underscores how the absence of risk recognition at the agenda-setting stage exemplified a failure to operationalize resilience as capability—adaptive, equity-attentive, and preparedness-driven. This omission represents the first rupture in the causal chain—one that critically shaped the structural vulnerabilities observed in Iran’s health system.

Sanctions during this period were primarily framed as diplomatic or geopolitical issues, falling within the remit of foreign policy rather than health governance. Such narrow framing entrenched siloed governance, leaving health policy institutions without the mandate or mechanisms to confront the structural impacts of sanctions (Vedadhir et al. 2025). Although upstream policy documents articulated these principles, they were not translated into operational mechanisms to enhance resilience to sanctions (Sajadi et al. 2020). This agenda-setting failure left Iran’s health system without a coherent or anticipatory strategy to confront one of the most consequential structural shocks of the contemporary era.

In the policy ‘formulation phase’, the second rupture occurs at the level of intervention design, where foundational assumptions were adopted without a formal risk appraisal. Although the HTP aimed to address financial deficits and inequities in access, it lacked structural coherence and omitted resilience-oriented risk assessments (Sajadi et al. 2019). While the plan outlined key services—such as referral systems and essential care—it rested on implicit assumptions of sustained financing, currency stability, and an intact insurance infrastructure, none of which were critically examined. These oversights created a fundamental misalignment between the plan’s stated objectives and the realities of Iran’s volatile policy and fiscal environment. Mechanisms to ensure accountability, enable intersectoral coordination, and support adaptive implementation remained either underdeveloped or absent (Sajadi et al. 2024a). This finding reflects contemporary shifts in policy analysis—from assessing basic capacity to evaluating institutional capabilities, such as strategic governance and adaptability in crisis contexts (Smith 2020, Witter et al. 2023).

The ‘implementation phase’ revealed a notable absence of coordinated planning and institutional readiness, particularly in managing external shocks and aligning operational responses with strategic objectives. Key interventions—such as pharmaceutical supply and emergency resource mobilization—were implemented without structured or forward-looking planning and therefore failed to develop the adaptive capacities essential for sustained resilience. These fragmented responses were unable to consistently alleviate medicine shortages, scale domestic production, or meaningfully reduce reliance on global supply chains—ultimately leaving vulnerable populations underserved. These shortcomings underscore both a moral and strategic imperative: that equity must be embedded at the core of any resilience-building agenda. Yet, as Saulnier and Topp caution, resilience is not inherently equitable—systems can adapt in ways that reinforce existing inequalities or shift the burden of adjustment onto vulnerable groups. Acknowledging the risk of ‘maladaptive resilience’ underscores the need to critically assess which resilience pathways are prioritized—and whose interests they ultimately serve (Saulnier and Topp 2024). Evidence from district-level health systems suggests that under chronic stress, service delivery is often sustained through local improvisation, informal networks, and relational problem-solving. Although this form of ‘everyday resilience’ may help absorb short-term shocks, it is inherently fragile in the absence of systemic support and institutional reform (Gilson et al. 2017). The Iran–Israel war provided a further real-time example of this dynamic, with communities mobilizing to compensate for institutional deficits and service disruptions (Sajadi and Majdzadeh 2025b). Community-led responses—including informal care coordination and neighbourhood-based support—played a pivotal role in sustaining essential services. These efforts exemplify the foundations of community resilience. Yet, while communities are increasingly absorbing the burden of crisis response, they remain under-supported—underscoring the urgent need for institutionalized strategies that enable, sustain, and scale grassroots models of community resilience. Translating local resilience into lasting system change requires policy mechanisms that formally embed community-led responses into national strategies. Without such integration, grassroots efforts risk remain fragmented and unsustainable.

As classified by Witter et al., Iran’s experience aligns with the fifth category of shocks: a complex blend of chronic stress—primarily from sustained international sanctions—compounded by acute socio-economic and health crises. Such conditions necessitate layered strategies that integrate shock absorption, adaptive response, and system-wide transformation (Witter et al. 2023).

Institutionally, resilient health systems are characterized by structural flexibility, transparent and accountable leadership, and agile decision-making—all of which were insufficiently institutionalized in Iran’s health sector response to sanctions (Al Asfoor et al. 2024). Empirical evidence illustrates the consequences of this institutional fragility, including severe reductions in access to essential medicines and basic services—particularly among marginalized populations—throughout the sanctions period (Sajadi et al. 2023). In several regions, these disruptions led to acute shortages, increased healthcare costs, and exacerbated health disparities (Sajadi and Majdzadeh 2022).

During the ‘evaluation phase’, the analysis identified the absence of structured learning mechanisms as a pivotal failure point. Applying the RLHS framework revealed that most policies lacked continuous mechanisms for generating real-time evidence and engaging stakeholders meaningfully (Ahmadi et al. 2024). These deficiencies, compounded by financial and logistical barriers, delayed vaccine procurement and compromised population-level protection (Sajadi and Majdzadeh 2022, Vedadhir et al. 2025). Moreover, the lack of transparency in humanitarian exemption mechanisms hampered Iran’s ability to respond effectively to the crisis (Karimi and Turkamani 2021). Over time, sanctions have also eroded national capacities for research, education, and innovation—critical dimensions that must be embedded in any comprehensive resilience strategy (Al Asfoor et al. 2024). The European Observatory notes that fostering a ‘culture of organizational learning’ is foundational to effective resilience, rooted in robust feedback loops, the use of empirical data, and evidence-informed decision-making (Thomas et al. 2020). In this context, the Resilience Testing framework designates ‘learning and recovery’ as the fourth stage of shock response, advocating adaptive assessments across four critical health system domains: governance, resources, service delivery, and financing (Zimmermann et al. 2024).

Building on the structural challenges outlined earlier and referencing the model presented in Fig. 3, this section assesses the extent to which Iran’s health policy architecture has internalized—or failed to internalize—resilience in the face of prolonged sanctions. This analytical lens exposes hidden assumptions, fragmented institutional trajectories, and structural blind spots that collectively undermined policy effectiveness. The model illustrates that Iran’s constrained resilience stemmed from broad structural weaknesses that became fully visible only under the pressure of compounded crises. Although grounded in Iran’s specific context, the architecture of Fig. 3 is conceptually anchored in an expanded ToC framework with broader applicability to health planning in other structurally constrained settings. By systematically tracing preconditions, assumptions, breakpoints, and root causes—while integrating recursive feedback—the model moves beyond descriptive mapping and offers a replicable diagnostic tool for resilience analysis. Crucially, the model incorporates the intersectoral character of resilience failures, acknowledging that critical breakdowns—in financing, supply logistics, or institutional continuity—frequently originate outside the formal boundaries of the health sector. In this sense, the model provides a broader conceptual framework for integrating resilience into national planning under prolonged political, economic, and intersectoral stress.

The model delineates a comprehensive causal pathway—beginning with key prerequisites such as sustainable financing, effective governance, and organizational learning. It then maps core assumptions (e.g. political stability, access to global markets), linked to interventions like the HTP and other sanction-response efforts, culminating in performance indicators and eventual points of failure, including pharmaceutical supply chain breakdowns and health-workforce attrition.

The ‘root cause analysis’ identified three principal drivers of this fragility: (i) the absence of structural risk diagnostics in policy design; (ii) a lack of institutionalized, real-time learning mechanisms; and (iii) external shocks—such as sanctions and pandemics—that exceeded the system’s absorptive capacity. These insights are consistent with theoretical perspectives that conceptualize resilience not as a static collection of capacities, but as an emergent property of dynamic interactions between system functions, actors, and institutions (Witter et al. 2023). The distinction between ‘capacity’ and ‘capability’ reinforces this point: financial resources alone are not sufficient; instead, resilience requires the institutional ability to adapt and make decisions under uncertainty (Zimmermann et al. 2024). Viewed through a social justice lens, resilience-oriented policy design must deliberately prioritize populations experiencing intersecting forms of vulnerability and marginalization (Vedadhir et al. 2023, Sajadi and Majdzadeh 2025a).

International comparisons help situate Iran’s challenges within a broader global context. For example, recent analyses from countries such as Thailand, Uganda, and Costa Rica demonstrate how co-ordinated, multisectoral responses facilitated more effective navigation of the COVID-19 crisis. They operationalized flexible governance models and harnessed public–private partnerships to strengthen intersectoral resilience (McClelland et al. 2023). In contrast, experiences from Iraq, North Korea, and Venezuela reveal how sanctions imposed on fragile economies with constrained institutional capacity can devastate public health—manifesting as disrupted financing, critical medicine shortages, and eroded governance structures (Pinna Pintor et al. 2023). Such cases underscore the imperative for adopting layered, resilience-driven policy frameworks in comparably vulnerable contexts—including Iran (Sajadi and Majdzadeh 2022, Sajadi et al. 2023, Vedadhir et al. 2025). Since several resilience barriers arise from external constraints—such as sanctions or restricted access to medical goods—some interventions inevitably lie beyond national control. The international-level recommendations proposed in this study reflect these structural realities and directly correspond to the challenges identified through our root cause analysis.

By synthesizing insights across the agenda-setting, policy formulation, implementation and evaluation phases, the framework revealed specific points of failure and institutional blind spots that collectively undermined resilience within Iran’s health policy architecture. The authors did not predefine the policy recommendations advanced here; instead, they emerged inductively through thematic synthesis of empirical data and were validated through expert interviews and Delphi rounds. Accordingly, they reflect both the policy priorities and perceived feasibility thresholds of stakeholders actively engaged in Iran’s health governance landscape.

International-level recommendations: This study highlights the urgent need for robust legal frameworks that ensure uninterrupted access to essential health services during sanctions. Although humanitarian exemptions theoretically exist, their practical ineffectiveness has been well-documented—particularly in the form of disrupted medical supply chains and blocked financial transactions. There is a compelling need to strengthen enforceable international legal instruments—especially those overseen by neutral global bodies—to safeguard the right to health amid the challenges of geopolitical constraints. Equally, such frameworks should incorporate accountability mechanisms that hold sanctioning entities responsible for the humanitarian consequences of their measures, thereby ensuring that the burden of protection does not fall solely on the affected health systems (Sajadi et al. 2025). Beyond legal protections, international financing arrangements must also reduce sanctions-induced fragility. Two priorities are to (i) advance reforms in the global financial architecture that expand fiscal space for health in sanctioned settings, and (ii) deploy innovative debt relief instruments—such as debt-to-health and debt-for-nature swaps—to redirect debt service towards health system strengthening (The Lancet Global Health 2025).

Another key vulnerability is the absence of protected, internationally coordinated supply corridors for critical medical goods. Establishing such humanitarian corridors is not a normative ideal, but an empirically grounded response to the documented failure of domestic mitigation mechanisms under compounded shocks. Absent these mechanisms, the health systems of sanctioned countries remain structurally vulnerable—regardless of the robustness of their internal reforms. In this context, the United Nations Special Rapporteur on the negative impact of sanctions on the enjoyment of human rights has launched a formal call for stakeholder submissions to inform the development of practical principles aimed at mitigating the humanitarian and health impacts of sanctions. This initiative seeks concrete, evidence-informed proposals from States, international organizations, civil society, and other stakeholders on ways to ensure unhindered humanitarian access in sanctioned contexts (United Nations Human Rights Council 2025).

National policy measures: At the national level, the most pressing deficit lies in the absence of risk-informed planning during the design stage of health policy. This omission has fostered an over-reliance on assumptions—such as stable financing or uninterrupted supply chains—that consistently collapse under sanction conditions. Domestic revenue and efficiency measures are crucial for sustaining core services under sanctions. Governments can broaden health financing by increasing the progressivity of taxation and by raising/earmarking excise taxes on tobacco, alcohol, and sugar-sweetened beverages for essential health packages. In tandem, tackling inefficiency—through anti-corruption initiatives and stronger public financial management—can recover resources for priority services without increasing the overall tax burden (The Lancet Global Health 2025). Integrating risk scenarios into policy formulation is essential for anticipating future shocks and institutionalizing adaptive governance.

Equally critical is the institutionalization of learning health systems—structures that embed real-time feedback loops into policy adjustment. Rapid feedback loops (dashboards, learning collaboratives, after-action reviews) should be embedded in budgeting and service delivery. Targeted investments in decision-support infrastructure will build institutional memory and long-term agility. Complementing these national measures, the UN Special Rapporteur has issued a call for inputs on methodologies to monitor and assess the humanitarian impacts of sanctions over time; such international monitoring can provide comparative benchmarks and support domestic policy learning (United Nations Human Rights Council 2025). From a normative standpoint, resilience must be anchored in social justice, inclusive governance, and equity—principles that are operational imperatives in fragile and unequal settings.

Collectively, these five policy directions—two international and three national—constitute layered and complementary responses to the root causes of resilience failure identified in this study. Rather than prescriptive end-goals, they serve as adaptive scaffolds designed to address structural vulnerabilities: institutional fragility, disrupted global connectivity, and underdeveloped learning architectures. To shift from reactive crisis management to anticipatory policy design, governments must prioritize capabilities over isolated capacities. These capabilities include strategic foresight, multi-level leadership, and localized, context-sensitive decision-making. As emphasized in the OECD Health Division’s resilience framework, such capabilities must be tested through structured, scenario-based exercises that move beyond traditional health indicators to encompass governance, institutional coordination, and systemic adaptability (Zimmermann et al. 2024).

This study employed a ToC framework to analyse structural weaknesses and reconstruct causal pathways within Iran’s health policy response to prolonged economic sanctions. ToC has gained increasing recognition as both a conceptual and practical tool for evaluating complex policy environments—particularly within health systems research and public sector reform (Rogers 2014, Mayne 2015, Breuer et al. 2016). Recent WHO guidance likewise endorses ToC as an appropriate approach for designing evidence-informed policy frameworks under conditions of uncertainty and systemic stress (World Health Organization 2024). Nonetheless, we acknowledge that relying exclusively on ToC introduces certain interpretive limitations and may affect the internal validity of our findings. While the framework was chosen for its diagnostic strengths—particularly in tracing assumptions, breakpoints, and feedback failures across the policy cycle—we did not undertake a comparative analysis using alternative approaches such as systems thinking, realist evaluation, or scenario-based modelling. As such, our analysis reflects both the strengths and constraints inherent to a single-framework methodology. We recommend that future research explore theoretical triangulation by incorporating complementary approaches and methods to deepen causal inference and enhance the methodological robustness of resilience diagnostics.

Caution is warranted when interpreting the findings of this study, given its contextual grounding in document analysis, qualitative data, and a Delphi process conducted entirely within Iran. While analytically robust, the insights may not be directly generalizable to other settings. Future research should triangulate ToC with realist evaluation, systems thinking, and scenario planning to deepen causal inference. While ToC provides a structured framework for tracing assumptions and causal pathways, systems thinking is more accurately described as an analytical approach that highlights interdependencies, feedback loops, and emergent properties within complex health systems. Likewise, scenario planning (sometimes supported by modelling techniques) should not be seen as a framework but rather as a foresight method that enables the exploration of multiple plausible futures under conditions of uncertainty. Integrating these perspectives with ToC would therefore allow future research to examine both the structural logics of policy design and the dynamic, context-dependent contingencies that shape resilience (Zimmermann et al. 2024). Another priority is to develop operational, data-driven models that produce continuous, objective, and cross-contextual measures of health system resilience (Al Asfoor et al. 2024). Future work should triangulate ToC with systems thinking, realist evaluation, and scenario modelling to deepen causal inference.

## Conclusion

The available evidence indicates limited progress in structural resilience-building during poly-crises (sanctions and COVID-19). Reported vulnerabilities relate not only to implementation challenges but also to underlying policy assumptions and early-stage structural design choices within the policy cycle.

In light of these findings, policymakers should move beyond isolated capacity-building to foster institutional capabilities—particularly in strategic governance, adaptive learning, and systemic responsiveness. Routinely applying structured resilience-testing frameworks can support anticipatory governance and promote incremental, evidence-informed reform.

Resilience should be understood not as mere continuity, but as the system’s capability to adapt, learn, and evolve under persistent stress. Future research should explore complementary approaches and methods, such as systems thinking or scenario modelling, to further examine resilience dynamics in politically constrained health systems.

Global resilience frameworks should therefore recognize economic sanctions as systemic threats—on par with pandemics and climate emergencies—to ensure that no population is rendered structurally vulnerable by geopolitical isolation.

## Data Availability

This study is a synthesis of several primary research projects conducted by the authors between 2014 and 2023, as listed in Table 1. As such, data availability varies across these underlying studies. Access to the data for any specific component should be sought through the corresponding primary study, each of which outlines its own conditions, ethical restrictions, and procedures for data sharing.

## Reflexivity statement

Both authors have worked within Iran’s academic health system and lived there during extended periods of economic sanctions. These experiences have shaped their professional engagements and their lived understanding of how sanctions impact health systems. They were affiliated with the National Institute for Health Research (NIHR) in Iran for several years, where they contributed to policy evaluation and implementation analysis across various health programmes. HS.S. holds a PhD in Health Services Management, while RM is a trained epidemiologist with a master’s in Health Technology Assessment.

The authors hold no institutional or political affiliations with any entities or policy actors that affect or impact the results of this study. While their proximity to the context provided in-depth access and contextual understanding, the final outputs of Phase two were validated through consultations with five independent health system experts in Iran, ensuring the plausibility and contextual relevance of the findings. The authors acknowledge that reflexivity is a continuous process and have aimed to present their analysis in a transparent and critical manner.
